# The Role of lncRNA in the Development of Tumors, including Breast Cancer

**DOI:** 10.3390/ijms22168427

**Published:** 2021-08-05

**Authors:** Beata Smolarz, Anna Zadrożna-Nowak, Hanna Romanowicz

**Affiliations:** 1 Laboratory of Cancer Genetics, Department of Pathology, Polish Mother’s Memorial Hospital Research Institute, Rzgowska 281/289, 93-338 Lodz, Poland; hanna-romanowicz@wp.pl; 2 Department of Chemotherapy, Copernicus Memorial Hospital, Medical University of Lodz, 93-513 Lodz, Poland; anna.m.zadrozna@gmail.com

**Keywords:** tumors, breast cancer, lncRNA, expression

## Abstract

Long noncoding RNAs (lncRNAs) are the largest groups of ribonucleic acids, but, despite the increasing amount of literature data, the least understood. Given the involvement of lncRNA in basic cellular processes, especially in the regulation of transcription, the role of these noncoding molecules seems to be of great importance for the proper functioning of the organism. Studies have shown a relationship between disturbed lncRNA expression and the pathogenesis of many diseases, including cancer. The present article presents a detailed review of the latest reports and data regarding the importance of lncRNA in the development of cancers, including breast carcinoma.

## 1. lncRNA—History

The history of lncRNA, or long noncoding RNA, dates back to the early 21st century. In 2001, two groundbreaking works were published—the first in Nature and the second in Science—which presented 96% [1] and 100% of the human genome, respectively [2]. The final sequence of the human genome was released in 2003. In what came as a surprise to the world of science, only a small percentage (1.2%) of human genetic material was found to encode proteins. The remaining ~99% are noncoding DNA, of which 24% are intron DNA and 75% are intergenic DNA [3].

In 2012, the ENCODE (Encyclopedia of DNA Elements) consortium showed that, despite only a small number of genes encoding proteins, human genetic material is 93% transcribed, of which 39% of transcripts correspond to introns and UTR sequences of protein-coding genes, 1% to exons, and 54% to noncoding genes [3]. These findings contributed to the development of interest in noncoding sequences and their transcripts, initially thought to be merely “junk DNA”.

The first long noncoding RNAs, treated at the time of discovery as mRNA, were the *H19* and *Xist* genes. The *H19* gene was discovered while studying the phenomenon of genomic imprinting. Genomic imprinting (parental genomic stigma) is associated with methylation and, consequently, the exclusion of a single maternal or paternal allele of a given gene in ova or sperm cells, respectively. This phenomenon, occurring during gametogenesis, preserves the variability of organisms [4]. The *H19* gene was identified in the 1980s on chromosome 7 of a mouse [5], where it formed a cluster together with the insulin-like growth factor gene *Igf2* [5,6]. The *H19* gene was inherited from the mother, and the *Igf2* gene was inherited from the father [3,5,6]. In contrast to the *Igf2* gene, which encodes the protein, the *H19* gene was transcribed but not translated [3].

The nucleotide sequence of the *H19* gene was conserved in mammalian genetic material. However, the described gene had mRNA features—being transcribed by RNA polymerase II, spliced, and located in the cytoplasm—and was initially recognized as such a molecule [3,7]. It garnered renewed interest from scientists after the discovery of another gene that does not encode a protein—the *Xist* gene. It is now known that the product of the *H19* gene is a suppressor of tumors.

The *Xist* gene belongs to a gene complex in a region of the X chromosome called the XIC (X-inactivation center). This complex is involved in the process of disabling one of the X chromosomes in women (or other female mammals), thus equalizing gene expression in women and men [8]. This process was first described by geneticist Mary Lyon and is often referred to, in her honor, as Lyonization or Lyon’s law [9]. The exclusion of one of the X chromosomes in a woman’s cells occurs at random during embryogenesis [8].

The *Xist* gene is crucial for the proper conduct of this phenomenon. In the first stage, it is expressed on the X chromosome, intended for inactivation. The product of the *Xist* gene—long noncoding RNA—then flattens the “selected” X chromosome, inducing the connection of subsequent factors (e.g., PCR2 complex), which leads to a change in chromatin conformation and, as a result of repression of most genes, formation of an inactive Barr body [3,8]. This function of disabling the entire chromosome is unique in the world of lncRNA.

## 2. lncRNA—Characteristics

Long noncoding RNA are molecules with a length of more than 200 base pairs. These molecules are transcribed by RNA polymerase II, occasionally by RNA polymerase III, and also, in the plant kingdom, by RNA polymerases IV and V [3]. Many lncRNAs have a 5′ cap, which makes their RNA structure more stable, with the exception of lncRNA derived from larger molecules (such as intronic lncRNA and circRNA) [3]. Stabilization of the lncRNA structure is also influenced by polyadenylation at the 3′ end, but this only occurs in certain parts of the molecule. Some lncRNAs may occur in both forms, i.e., either with or without a polyadenylated 3′ end (known as bimorphic lncRNA) [3].

Long noncoding RNAs contain many exon regions, which allow for the creation of diverse forms of this RNA family as a result of splicing. These diverse forms may perform different functions, including those of clinical importance [3]. In addition, the genes of these noncoding ribonucleic acids have a structure similar to protein-coding genes (PCGs), but the level of lncRNA gene expression is much lower. Lower expression may be due to the difference in the structure of lncRNA gene promoters and enhancers (especially in the context of epigenetic histone changes), which reduces the severity of the transcription process and results in lower stability of the lncRNA molecule compared with the mRNA molecule [3].

The stability of lncRNA molecules depends on their type. The lncRNA intron and promoter-related lncRNA are less stable than the intergenic, antisense, or end-related 3′ UTR [3,10].

Single-exon transcripts (a type of nuclear lncRNA) are considered unstable. In human cells, antisense lncRNA has been shown to be more stable than mRNA (half-lives of 3.9 vs. 3.2 h, respectively), and intronic lncRNAs have the form of both stable transcripts (with half-lives above 3 h) and unstable transcripts (t1/2 < 1 h), with an average half-life of 2.1 h [3,10]. The recently discovered circular RNA (circRNA) is a highly stable molecule with a half-life of approximately 19–24 h [3,11]. However, half-life times are estimates, due to the fact that lncRNA is very susceptible to cellular stress, so these times may vary depending on the conditions of the experiment. In vivo testing is necessary [3].

The expression of specific lncRNAs is characteristic of individual tissues and even cells [3,12]. Only a small number of lncRNAs are common throughout the body (e.g., MALAT1); however, these are usually found in high concentrations [13]. Long noncoding RNAs typical of tissues or cells show significantly lower expression [13]. Studies have shown that diseases associated with single-nucleotide polymorphisms within lncRNA genes and their promoters are associated with a changed expression of these lncRNAs, which would confirm their importance in disease pathogenesis. In addition, the specificity of lncRNA expression can testify to their key role in regulating the functioning of organisms, as well as in repairing pathological processes [14].

Long noncoding RNAs, unlike mRNA, are found in the cell nuclei, cytoplasm, and mitochondria; simultaneously in the nuclei and cytoplasm; only in the nuclei; or only in the cytoplasm [15]. Thanks to this distribution, lncRNAs are able to perform a variety of functions affecting mRNA stability, translation, and cell signaling pathways. Nuclear lncRNAs also perform functions affecting chromatin, transcription, RNA treatment, and the cytoplasm. Changes in the environment or infections can force lncRNAs from one cellular component to another [15]. In addition, lncRNA is a dynamic, adaptable molecule that can take a secondary structure. Thanks to its flexible design and the versatility of lncRNA functions, specific cellular decomposition—particularly in the nucleus—as well as interaction with proteins are made possible [3]. The conformation of lncRNAs allows them to avoid the evolutionary limitation of poor interspecies conservation. On the other hand, low conservation of the lncRNA sequence likely enables the variability of the structure, and with it, the function and specialization lncRNAs as a regulator [3,16].

**Figure 1:**
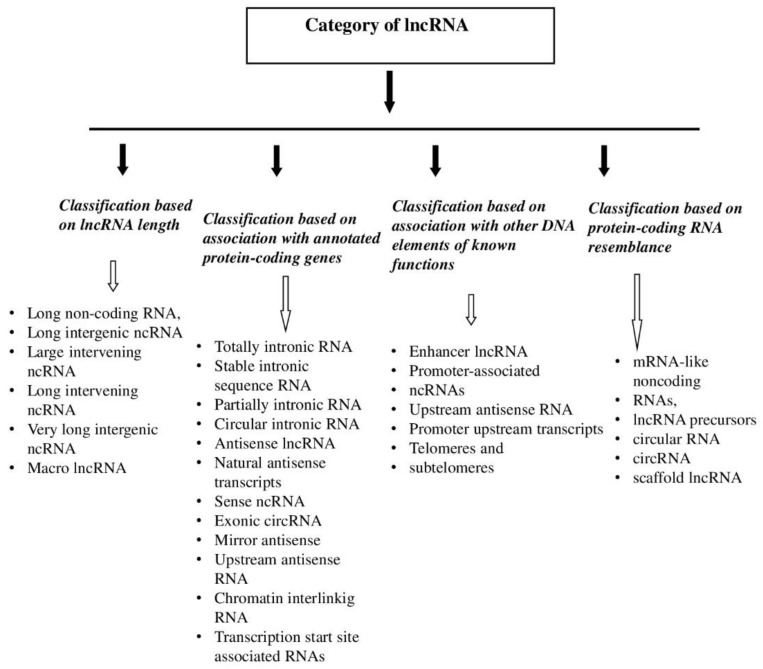
Classification of lncRNA.

Long noncoding RNAs are the largest group of ribonucleic acids and remain the least understood. Figure 1 presents the classification of lncRNA. It seems important to divide the classes by genome location; however, to understand the functions of lncRNAs, it is easier to distinguish them into cis- and trans-interacting classes (according to Kopp, F. and Mendell, J.) [17].

## 3. lncRNA—Functions

The cis method indicates the effect of lncRNA on the expression of adjacent genes. The best example of cis action is the Xist transcript function described above [17]. The demonstration of gene expression, in this case the silencing of the entire chromosome, is evidently influenced by the transcript itself inducing the connection of protein complexes. However, cis regulation can occur independently of the lncRNA transcript.

In some cases, as in the case of Igfr2 paternal silencing, the overlap of the Airn sequence (antisense Igfr2 RNA noncoding) with the Igfr2 gene promoter has an impact on the adjacent gene, which impairs the connection of RNA Polymerase II and, as a result, the transcription process [17,18].

In a study by Latos et al., it was shown that a full Airn transcript is not needed to switch off the Igfr2 gene, but only an antisense sequence complementary to the Igfr2 gene promoter [18].

In contrast, Eingreitz et al. demonstrated that the cis regulation of the *Sfmbt2* gene was dependent not on the preserved sequence of Blustr lncRNA, but on the length of its transcription (the longer the transcription activity in the Blustr locus, the greater the expression of Sfmbt2), as well as the splicing of Blustr associated with the 5′ end [17,19]. The authors of the study assume that the altered transcription or splicing changes the chromatin conformation in the promoter region of the *Sfmbt2* gene and weakens the connection to the RNA polymerase promoter.

Sigova et al. described another mechanism of the influence of lncRNA on gene expression, this time through so-called trapping of transcription factors [20]. The authors noted that transcription factors can attach to both proximal promoter sequences and distal transcribed DNA elements, as well as the resulting RNA. In their study, the authors demonstrated that the transcription factor YY1 (YingYang1) attaches to DNA regulators and to RNA [20]. Reducing transcription resulted in YY1 being suppressed, and RNA connection increased YY1 activity in regulatory regions. Thus, it was suggested that RNA affects the function of transcription factors on a feedback basis and contributes moderately to the regulation of gene expression [17,20].

Another concept of cis regulation, as yet supported by little evidence [17,19], assumes that the DNA elements of the lncRNA gene are responsible for regulating the expression of adjacent genes. In the Eingreitz et al. study cited by Kopp and Mendell, decreased expression of adjacent genes for the lncRNA studied was demonstrated after several of its promoters had been removed. Reduced expression of adjacent genes was not achieved during lncRNA transcription at an earlier stage [17].

To summarize the above-described mechanisms of gene expression regulation in the cis manner, lncRNA affects the transcription process directly, acting as an enhancer, by “stopping” transcription factors, and by affecting chromatin looping and gene methylation (using epigenetic complexes such as PCR2) [21].

Trans regulation, in turn, is about controlling the expression of distant genes. Long noncoding RNAs can regulate these genes by affecting their promoters and enhancers, or via proteins associated with these regions, and, together with the attached proteins, by affecting chromatin conformation and polymerase activity [17].

Some lncRNAs are elements of complexes necessary for transcription or splicing. By facilitating the transport of these structures to the areas of the transcribed genes, they affect the structure of the cell nucleus [17]. In addition, lncRNAs bind proteins that combine with RNAs or RNA itself, e.g., microRNAs [17]. They regulate not only transcription, but also the post-transcription processes. Two of the first transfunctional lncRNAs discovered were the HOTAIR and MALAT1 transcripts, which, as further research has shown, play a significant role in the carcinogenesis process.

## 4. lncRNA and Malignant Tumors

The characteristics of long noncoded RNA described above—such as tissue or cellular specificity and the regulation of gene expression at the transcriptional and post-transcriptional levels—indicate that lncRNAs may be important in the formation of malignant tumors. Studies have shown that lncRNAs affect the pathways of division, growth, and cell differentiation, and are also involved in cellular death processes [21,22]. Modifications to these processes may lead to carcinogenesis [22]. Moreover, some lncRNAs are regulated by oncogene products or cancer transformation suppressors, which means that they are believed to indirectly perform tumorigenic functions (Table 1) [22].

Modern sequencing methods revealed different expressions of individual lncRNAs in cancerous tissues compared with healthy tissues [23]. The first observed transcripts of altered expression in the tissue of a malignant tumor—prostate cancer—were PCA3 and PCGEM1. PCA3 currently functions as a cancer marker [22]. The previously mentioned MALAT1 was also discovered in cancerous tissue as one of the first lncRNAs. Of prognostic significance, its altered expression was discovered in the tissues of lung cancer [24]. It is now known that altered expression of this transcript occurs in many cancers, which may indicate its importance in the process of cellular proliferation [25].

Altered lncRNA expression is not the only disorder of these molecules that occurs in cancerous tissues. Many lncRNA genes have been found in regions where somatic changes in the number of DNA copies (SNCA) occur in the form of deletion or amplification. These aberrations are extremely common in cancer cells [22]. Long noncoding RNAs also encounter single-nucleotide polymorphisms [22], and their transcription is regulated by factors that control the main cellular processes of homeostasis—including oncogenes and tumor suppressors—with the best examples being p53 and MYC factors.

DNA damage, a specific accumulation of which is observed in cancer cells, causes activation of the transcription factor p53. This factor, depending on the degree of DNA damage, induces apoptosis or halts the cell cycle for the duration of repair. One element of the proapoptotic pathway or the suspension of cell division is an increase in lncRNA transcription through the p53 factor.

The resulting transcripts are involved in the regulation of these pathways and thus modulate responses to cellular stress [22,26,27]. On the other hand, some lncRNAs affect p53 function by interacting with the gene enhancers of this protein [28]. Finally, the MEG3 transcript—a reduced expression of which has been found in many malignancies [22]—activates factor p53 itself [26,29].

The MYC transcription factor is an oncogene involved in the processes of cell proliferation, metabolism, and growth; angiogenesis; and metastasis. The transcriptionally active region of the MYC locus is one of the most commonly amplified in malignant neoplasms [22].

There are also many lncRNA genes in the region that, like the MYC oncogene, are translated more intensively [22]. Some of these lncRNAs regulate expression of the MYC gene in a cis manner [22]. On the other hand, expression of lncRNAs from the described region, involved in repression of the genes regulating the cell cycle according to MYC, is modified by the aforementioned proto-oncogene [22,30,31].

Studies have shown that the expression of oncogene and tumor transformation suppressors in malignant tumor tissues is increased and decreased, respectively [32].

This suggests that the interaction between lncRNAs and oncogenes or tumor suppressors is an important mechanism that contributes to the initiation of carcinogenesis. Another important mechanism seems to be the effect of lncRNAs on chromatin-modulating complexes through epigenetic changes, as an accumulation of epigenetic DNA modifications is very common in malignant tumors [33]. A well-understood epigenetic complex is PCR2 (polycomb repressive complex 2), which reduces gene expression by trimming histones (H3K27). Research indicates that many long noncoding RNAs affects PCR2 function [34].

An example of a lncRNA connecting to the PCR2 complex is the aforementioned trans-acting HOTAIR. Overexpression of this transcript occurs in many malignant tumors [35] including breast cancer [36], and is associated with poor prognosis. HOTAIR hyperactive complex with PCR2 causes increased gene suppression, contributing to the formation of metastases [37]. The mechanism described is an example of the agonist action of lncRNAs and chromatin modulating complexes. Some lncRNAs also exert antagonistic effects [22].

Long noncoding RNA can also affect cell homeostasis by post-transcriptional regulation; mRNA splicing, processing, and translation; or post-translational protein modification [22]. In addition, it has an impact on mRNA by binding microRNAs, reducing the amount of free short RNA and thereby reducing their impact on encoding transcripts [22].

The proper functioning of the cell is conditioned by the balance of metabolic processes. Studies show that lncRNAs are involved in the basic pathways of cellular metabolism, including in cancer cells, e.g., in the production of ATP under hypoxic conditions via HIF-1alpha factor or the Warburg effect [26].

A characteristic feature of malignant tumors is the ability to bypass immune control. Reports indicate that lncRNAs are involved in regulating the immune response by modifying the activity of immunocompetent cells [38]. For this reason, lncRNAs appear likely to be involved in the formation of immunomicelles of malignant tumors, even improving their defenses against the immune system [26,39].

It was mentioned above that overexpression of the HOTAIR transcript, which connects to the PCR2 complex, contributes to the formation of metastasis. Long noncoding RNAs also support the metastatic process by participating in epithelial–mesenchymal transition (EMT), as well as in signaling pathways associated with the activation of cancer stem cells [26]. Given the importance of lncRNAs in carcinogenesis, as well as the proven altered expression of these molecules in the tissue of malignant tumors, it seems natural to assess whether lncRNAs may represent new predictive factors. Over the past decade, much research has been conducted to analyze this problem, primarily in China.

In 2016, Serghiou et al. published a systematic review and meta-analysis of 111 studies assessing the effects of different lncRNAs on prognosis in malignancies: 83% of studies determined the effect of lncRNA on overall survival (OS) of patients, 32% on recurrence-free survival (RFS), 9% on disease-specific survival (DSS), 8% on metastasis-free survival (MFS), and 5% on progression-free survival (PFS) [40]. The study looked at 18 malignancies (almost half of which were gastrointestinal malignancies), primarily gastric cancer (16 studies), colorectal cancer (15 studies), and lung cancer (15 studies) [40].

In 96% of the studies analyzed, there was a statistically significant relationship between lncRNA expression and prognosis in malignancies. The vast majority of studies concerned HOTAIR and MALAT1 transcripts, the overexpression of which was associated with worse prognoses [40]. However, the authors of the publication point out that their work had many limitations.

## 5. lncRNA and Breast Cancer

Systematic reviews and meta-analyses were also conducted to assess the effect of altered lncRNA expression on prognosis in breast cancer alone.

Tian et al. analyzed 70 publications that examined the prognostic significance of the expression of 48 transcripts present in breast cancer tissues (in single blood tests) of 9307 patients [41].

The most commonly rated lncRNAs were MALAT1, HOTAIR, CCAT2, and MEG3.

In addition to the effect of lncRNAs on prognosis, the relationship between lncRNA expression and individual clinicopathological factors of breast cancer was assessed (Table 2).

This meta-analysis indicated that overexpression of CCAT2, MALAT1, and NEAT1 transcripts was associated with a shorter overall survival (OS) (hazard ratio, HR = 1.29, 95% CI: 1.03–1.63, *p* = 0.03; HR = 2.78, 95% CI: 1.95–3.97, *p* < 0.01; and HR = 1.65, 95% CI: 1.08–2.54, *p* = 0.02, respectively), and MEG3 transcript overexpression was associated with longer OS (HR = 0.47, 95% CI: 0.37–0.71, *p* < 0.01) [41].

A correlation was also seen between CCAT2 and HOTAIR overexpression and shorter metastasis-free time (MFS) (respectively, HR = 1.18, 95% CI: 1.02–1.36, *p* = 0.03; and HR = 1.90, 95% CI: 1.41–2.55, *p* < 0.01) [41]. Of the remaining lncRNAs, altered expression of 24 transcripts had an impact on OS; overexpression of seven of them (FGF14-AS2, AFAP1-AS1, EPB41L4A-AS2, BC040587, EGOT, GAS6-AS1, and FENDRR) was associated with better OS, while overexpression of the remaining 17 (BCAR4, HOTTIP, CCAT1, Z38, TUNAR, CRNDE, HULC, MVIH, TP73-AS1, linc-ITGB1, PVT1, UCA1, OR3A4, DANCR, LINP1, SNHG15, and SUMO1P3) was associated with worse prognosis [42]. In addition, the expression of nine transcripts (MALAT1, HOTTIP, MVIH, LINC00978, linc-ITGB1, MEG3, GAS6-AS1, HOTAIR, and LINP1) had an impact on DFS, MALAT1, MEG3 and HOTAIR expression on RFS, CCAT1, MEG3 and FENDRR expression on PFS and BCAR4 expression on MFS [41].

Only ten transcripts (MALAT1, MEG3, CCAT2, BCAR, TUSC7, TP73-AS1, NEAT1, TUG1, HOTAIR, and CRNDE) were evaluated in the meta-analysis in the context of clinicopathological factors.

This analysis showed that MALAT1 overexpression was significantly correlated with the presence of a progesterone receptor, and TUSC7 overexpression with HER2 receptor [41]. MEG3 overexpression was associated with a lower histological grade (G) malignancy, overexpression of NEAT1 and TP73-AS1, and with a higher stage of breast cancer according to the TNM classification [41] (Table 2). Of the remaining 38 transcripts studied, only four transcripts did not in any way affect the presence of clinicopathological agents [41].

The latest literature data indicate that lncRNAs are transcribed from cancer risk loci and that these transcripts can play important roles in tumorigenesis [42,43]. Betts et al. used RNA sequences to identify two estrogen-regulated lncRNAs (CUPID1 and CUPID2) that might contribute to the risk of developing breast cancer by modulating pathway selection of double-strand break repair (DSB) [44]. Studies demonstrated that CUPID1 and CUPID2 are important to homologous recombinational repair (HRR). Repair by recombination enables removal of a number of serious DNA damages including, first of all, double-strand breaks. These breaks may cause a loss of some chromosomes and induce translocation of genetic material between them. Moreover, they are strong inducers of programmed cell death. As demonstrated in the work of Betts and colleagues, the loss of CUPID1 and CUPID2 expression results in reduced DNA end resection and defects in pRPA and RAD51 recruitment, leading to reduced HR DNA repair, favoring 53BP1 recruitment and selecting NHEJ as a DNA repair pathway. The role of BRCA1 and 53BP1 and related partners in selection of a DNA repair pathway remains to be elucidated [45].

The credible causal variants (CCVs) at 11q13 fall within estrogen-regulated enhancers of two lncRNAs: CUPID1 and CUPID2. Fachal et al. defined CCVs within each signal (7394CCVs/196). CCVs are located in noncoding genomic regions [46] and reduce CUPID1/2 expression by inhibiting chromatin looping. CUPID1/2 play a role in modulating the choice of the pathway for repairing of double-stranded DNA breaks by promoting repair based on homologous recombination, providing a reliable mechanism by which CCVs alter the risk of breast cancer.

Marjaneh et al. demonstrated that CCV is enriched with exons but not in introns or mencRNA promoters, suggesting that genetic variants may alter the structure and/or function of mencRNAs [47].

Breast cancer is a heterogeneous disease with at least five molecular subtypes, including luminal A, luminal B, basal-like, HER2-enriched, and normal-like. These five molecular subtypes are usually stratified according to their mRNA profile patterns; however, lncRNA is increasingly used for this purpose.

NORAD and HCG11 are highly similar lncRNAs that contain binding sites for PUMILIO proteins. PUMILIO acts on hundreds of target mRNAs, helping to modulate gene expression. Mathias et al. analyzed NORAD and HCG11 expression levels in luminal A and basal-like breast cancer subtypes and the regulatory networks associated with these lncRNAs [48]. NORAD was upregulated in luminal A, while HCG11 was upregulated in the basal-like subtype. An increased NORAD expression is associated with reduced disease-free survival in basal-like patients, suggesting that its prognostic value may vary from subtype to subtype. The biological pathways observed for the HCG11 network are associated with epithelial–mesenchymal transition. NORAD-related pathways appear to be associated with the transformation of luminal epithelial cells.

Mathias and colleagues revealed that LINC01871 is associated with activation of the immune response and favorable overall survival in basal-like samples. EBLN3P is associated with immune response suppression and progression in the luminal B subtype, and MEG3, XXYLT1-AS2, and LINC02613 are associated with activation of immune response in luminal A, HER2-enriched, and normal-like subtypes, respectively. Further research is needed to better understand the role of lncRNAs as regulators of the immune response to provide new perspectives on diagnosis, prognosis, and therapeutic targets for the molecular subtypes of BRCA [49].

The latest scientific reports indicate that psoriasis-susceptibility-related RNA gene induced by stress (PRINS) is a novel lncRNA which is underexpressed in breast cancer in the Cancer Genome Atlas (TCGA). Chehade et al. showed that PRINS is underexpressed in primary breast cancer relative to normal breast tissues. Further research to investigate its molecular and intracellular effects is warranted to establish PRINS as a biomarker of functional importance in breast cancer [50].

Long noncoding RNAs are becoming understood as key players in the pathogenesis of cancer. Many lncRNAs have been shown to play a direct role in cancer cell proliferation, cancer progression, and/or metastasis. The specific tissue expression of lncRNA makes them exciting candidates for the development of diagnostic markers or presumed therapeutic targets for systemic treatment. The ability to target lncRNAs at different functional levels provides a wide range of therapeutic options. Targeted approaches may include nucleic acid-based drugs, small molecule inhibitors, and gene-editing methods. Targeted methods based on RNA are evolving rapidly to potentially cure various pathologies. Recent clinical success and FDA approval of antisense drugs for spinal muscular atrophy and Duchenne muscular dystrophy have led to various preclinical studies targeting lncRNAs using nucleic acid-based therapies. Further functional studies using appropriate preclinical models will confirm the importance of several lncRNA species in cancer pathogenesis, leading to a broad examination of this class of molecules as viable therapeutic targets in many types of cancer [51].

## 6. lncRNA—T-UCRs

Of those lncRNAs whose expression is altered in the tissues of malignant tumors, including breast cancer, some transcripts arise from so-called ultraconservative regions (UCRs). UCRs are genomic sequences that have survived evolution and are 100% compatible between the orthologous regions of humans, mice, and rats [3,52]. Of the 481 UCRs discovered, 111 coincide with sequences of genes encoding a human protein (exonic UCRs), 256 bear no resemblance to either the coding sequence or the resulting mRNA (nonexonic UCRs), and, for the remaining 114, insufficient evidence has been obtained to determine whether they are transcribed (possibly exonic UCRs) [52]. Of all known UCRs, 39% are intergenically located, 43% are found in intron sequences (including one hundred nonexonic UCRs), and 15% are in exon sequences [3]. Nonexonic UCRs, both intronic and intergenic, often form clusters near the genes of transcription factors and developmental proteins, but are also often found in so-called “gene deserts”, i.e., long, noncoding sections of DNA [53].

The importance of UCRs is not fully understood, but Bejerano et al. indicated possible functions of transcriptional regulators, including alternative splicing, RNA and DNA binding, and regulation of distal gene enhancers associated with development [52]. Jarroux et al. give an example of a transcript T-UCR (Evf2) that behaves like a decoy for the DLX1 transcription factor, as well as for a chromatin-modulating complex similar to the SWI-/SNF BRG1 gene, eventually withholding transcription [3].

## 7. T-UCRs and Malignant Tumors

The importance of T-UCRs in the process of carcinogenesis also remains unknown, although they are associated with oncogenes and suppressors of tumor transformation; participate in the apoptosis, proliferation, and migration of cancer cells; and also seem to regulate microRNA functions [43,54].

Moreover, studies have shown that transcription of certain T-UCRs is induced by hypoxia [55]. Despite the lack of relevant literature, there is evidence of a changed expression of T-UCRs in malignant tumor tissues.

One of the first studies confirming the presence of a changed number, compared with healthy tissues, of numerous T-UCRs in cancerous tissues—including cancer cells of chronic lymphocytic leukemia and tissues of colorectal and hepatocellular carcinoma—was that of Calin et al., published in 2007 [53,55]. In 2010, a changed expression of T-UCRs was also discovered in neuroblastoma tissues [56].

In 2012, a reduced amount of UC.73 and UC.388 was shown in the tissues of colorectal cancer, indicating the likely prognostic significance of these transcripts [57]. Subsequent years brought further evidence of deregulation of transcription of ultraconservative regions in prostate cancer [58], pancreatic cancer [59], cervical cancer [60], bladder cancer [61,62], gastric cancer [63,64], and lung cancer [65]. In the case of breast cancer, only two studies have been developed to analyze the expression and prognostic significance of T-UCRs—specifically, uc.63 and uc.38. The authors of the other three publications focus on determining the importance of single-nucleotide polymorphisms in ultraconservative regions and lncRNA genes.

## 8. T-UCRs and Breast Cancer

Marini et al. (2017) published a paper in which they determined uc.63 lncRNA expression in cultured breast cancer cell lines, compared with that of healthy breast tissue. Uc.63 is an ultraconserved region located in the third intron of the exportin 1 (*XPO1*) gene—a protein that exports certain molecules from the nucleus to the cytoplasm [66].

Transcription of uc.63 is induced by hypoxia [55]. The authors of the study observed a significant variety of uc.63 expression profiles in breast cancer cell lines. After rejecting the lines with low and very high expression of the study transcript, a cell line with high transcript concentration, from advanced breast cancer, was selected. In order to determine the probable biological function of the uc.63 transcript, silencing of the studied region was performed in the selected cell line.

The exclusion of uc.63 led to an increase in cancer cell death, which was diagnosed after observation of an increased number of cells in the G0/G1 phase, a reduction of cells in the G2/M phase, and detection of an increased concentration of the markers of apoptosis, PARP-1 and caspase-3 [66]. The above observations may indicate the importance of uc.63 in regulating the cycle of breast cancer cells [66]. The authors also point out that deregulation of *XPO1* gene transcription did not affect either uc.63 expression or cancer cell function [66].

To determine the prognostic significance of the altered expression of uc.63 in this malignant tumor, the authors performed bioinformatic analysis of the data of more than 2000 breast cancer samples from the Cancer Genome Atlas portal.

This analysis concluded that overexpression of uc.63 is associated with a reduction in disease-free time (DFS) in the luminal subtype A of breast cancer, but only in its more aggressive form [66].

Again, this correlation was independent of xpo1 gene expression [66].

In conclusion, the authors of the study suggest that uc.63 may act as an oncogene, and its overexpression may contribute to the survival and growth of breast cancer cells [66]. In addition, the authors point to the potential function of uc.63 as a predictor in luminal A breast cancer [66].

In a study by Zhang et al., uc.38 expression was evaluated in 100 breast cancer tissue samples and breast cancer cell lines, compared with normal tissues and cells of normal breast epithelium, respectively. This study showed that uc.38 expression was significantly reduced in cancer tissues [67]. As a result of the analysis of the relationship between the expression level of uc.38 and clinical and pathological factors, a correlation was observed between the reduced concentration of the uc.38 transcript and the higher stage of the cancer in question, according to TNM classification and the larger diameter of the primary tumor [67].

The above study also revealed a reduced expression of uc.38 in breast cancer cell lines compared with normal breast epithelial cells, which, the authors believe, may indicate an important role of uc.38 in the formation of the primary tumor and its progression [67].

An in vitro analysis was then carried out, which looked at the effect of increased or decreased expression of uc.38 on the breast cancer cell cycle.

This analysis noted that overexpression of uc.38 was associated with a significant reduction in cell proliferation, a decrease in the ability of cells to form colonies, and an increase in the number of cells subjected to apoptosis [67]. In contrast, reduced expression uc.38 had the opposite effect—increased proliferation, greater colony formation, and fewer apoptotic cells [67].

The evaluation of uc.38 expression and its consequences, as described above, are part one of a two-part study by Zhang et al.

In part two, the authors focused on the alleged regulation of the *PBX1* (pre-B-cell leukemia homeobox 1) gene by the lncRNA uc.38. PBX1 is a transcription factor whose overexpression is associated with poor prognosis in luminal breast cancers and promotion of metastasis [68]. This study showed that overexpression of uc.38 was accompanied by a lower concentration of PBX1 protein, and a reduced expression of uc.38 with an increased concentration of PBX1 protein [67].

The above data suggest that uc.38 controls PBX1 expression at the post-transcriptional translation level [67]. Further analyses indicated that the uc.38 transcript was present mainly in the cell nucleus, which, according to the authors, may demonstrate the role of uc.38 in regulating gene expression at the nuclear level [67].

As a result of RNA immunoprecipitation with PBX1 antibodies, an interaction between this transcription factor and lncRNA was demonstrated. Subsequent experiments demonstrated the dynamics of change in PBX1 protein concentration depending on the level of uc.38 expression: The silencing of lncRNA transcription resulted in an increase in PBX1 concentration [67]. Based on the above observations, the authors concluded that the transcript uc.38 not only had an effect on the PBX1 transcription factor, but also accelerated its degradation when uc.38 was present at an elevated concentration [67]. This relationship is an important part of the cell cycle; further studies demonstrated that PBX1 overexpression fosters the proliferation of breast cancer cells and halts the apoptotic process. The silencing effect of lncRNA uc.38 overexpression on the PBX1 transcription factor in turn contributed to cellular death induction [67]. The authors of this publication also analyzed the effects of uc.38 overexpression on breast cancer in vivo. After 4 weeks of follow-up, primary tumors were found in mice with uc.38 overexpression to have significantly lower volume and weight compared with primary tumors in control mice. In addition, the mice in the study group had significantly longer overall survival (OS) [67].

## 9. Conclusive Summary

Recent studies confirm assumptions about the great importance of both ultraconservative regions and other types of lncRNAs in pathogenesis and the course of malignant tumors, including breast cancer. The diagnostic, predictive, and even therapeutic potential of these unique molecules seems undeniable. While previously considered to be noncoding, they are in fact a matrix for the synthesis of short peptides [69]. These peptides are encoded in the sequence of short open reading frames (sORF) of lncRNA genes [70]. Recent reports indicate that these peptides play a significant role in the carcinogenesis process, providing new hope for breakthroughs in the search for effective anticancer treatment [71].

## Figures and Tables

**Table 1:** Long noncoding RNAs involved in cancer.

lncRNA	Genomic Location	Expression in Patients	Function in Tumorigenesis
PCGEM1	2q32.2	Increased in prostate cancer	oncogene
MALAT1	11q13.1	Increased in colon, lung, and liver cancers	oncogene
MEG3	14q32.2	Down-regulated in multiple cancers	tumor suppressor
HOTAIR	12q13.13	Increased in primary breast tumors and metastases, GIST, and pancreatic cancers	oncogene

**Table 2:** Summary of lncRNAs related to clinicopathological features and survival of breast carcinoma.

Clinicopathological Features	lncRNA
PR status	MALAT1
HER status	TUSC7
Histological grade	MEG3
TNM stage	NEAT1, TP73-AS1
**survival**	-
shorter overall survival	CCAT2, MALAT1, NEAT1
longer overall survival	MEG3
overall survival (better prognosis)	FGF14-AS2, AFAP1-AS1, EPB41L4A-AS2, BC040587, EGOT, GAS6-AS1, FENDRR
overall survival (worse prognosis)	BCAR4, HOTTIP, CCAT1, Z38, TUNAR, CRNDE, HULC, MVIH, TP73-AS1, linc-ITGB1, PVT1, UCA1, OR3A4, DANCR, LINP1, SNHG15, SUMO1P3
metastasis-free survival	CCAT2, HOTAIR, BCAR4
disease-free survival	MALAT1, HOTTIP, MVIH, LINC00978, linc-ITGB1, MEG3, GAS6-AS1, HOTAIR, LINP1
progression-free survival	MALAT1, MEG3, HOTAIR, RFS, CCAT1, MEG3, FENDRR

## Data Availability

Data sharing is not applicable to this article.
